# No association of apolipoprotein B gene polymorphism and blood lipids in obese Egyptian subjects

**DOI:** 10.1186/s12952-015-0026-8

**Published:** 2015-03-18

**Authors:** Neda M Bogari, Azza M Abdel-Latif, Maha A Hassan, Abeer Ramadan, Ahmed Fawzy

**Affiliations:** Faculty of Medicine, Department of Medical Genetics, Umm Al-Qura University, Makkah, Kingdom of Saudi Arabia; Division of Human Genetics & Genome Researches, Department of Molecular Genetics and Enzymology, National Research Centre, 33 Bohouth St. Dokki, Giza, Egypt; Holding Company for Biological products and Vaccines (VACSERA-Egypt), Cairo, Egypt

## Abstract

**Background:**

Several environmental and genetic factors are associated with high levels of lipids in obese patients. Apolipoprotein B (ApoB) is the major protein component of low-density lipoproteins (LDL), very-low density lipoproteins (VLDL) and chylomicrons and plays a central role in lipid metabolism. Several *apoB* restriction fragment length polymorphisms (XbaI, EcoRI, MspI) have been reported to be associated with variation in lipid levels and obesity. To date, no data are available on the relationship between XbaI polymorphism and lipid levels in Egyptian populations. Following clinical profiling, 178 obese (body mass index [BMI] >25 kg/m^2^) and 178 age-matched non-obese (BMI ≤ 25 kg/m2) subjects were included in this case–control study. All samples were analysed for total cholesterol, triglycerides and HDL-cholesterol. Genetic analysis of *apoB* XbaI (X) was performed using Polymerase Chain Reaction-Restriction Fragment Length polymorphism (PCR-RFLP). The aim of this study was to assess the association of *apoB* XbaI gene polymorphism (X) and lipid profiles in obese and non-obese Egyptian populations.

**Results:**

Obese subjects demonstrated significantly higher values of waist-to-hip ratio, blood pressure, and total lipid. However, in our sample we did not find significant differences in *apoB* XbaI gene polymorphism (X) genotype or allele frequencies. Moreover, none of the studied lipid parameters showed any association with the gene polymorphism.

**Conclusion:**

This study reveals no significant association of *apoB* XbaI gene polymorphism (X) with obesity or lipid profiles in an Egyptian population.

## Introduction

Obesity is a common, preventable and often underestimated, condition of clinical and public health importance in many countries. In many societies, including Egypt, obesity has been viewed as a sign of wellbeing or high social status. This has effectively led to the denial, by (generally high status) health care professionals and the public, that obesity is a disease. This has contributed to improper identification and management of obesity and the lack of effective public health strategies to combat its rise to epidemic proportions in all age groups [1]. *“The most shocking thing is the degree to which obesity is now affecting developing as well as developed economies”,* says Tim Lobstein of the International Association for the Study of Obesity in London. *“The problems caused by over consumption of fats and sugars are now global, not just Western, problems”.* The World Health Organization (WHO) reported that worldwide obesity has nearly doubled since 1980. The increasing prevalence of obesity places a large burden on health care use and costs. In 2006, WHO predicted that obesity in the developing world would overtake that in rich countries by 2010.

The situation in Egypt confirms the WHO prediction. Nearly 70% of the adult population – about 56.5 million people – are overweight, according to a new study published in The Lancet [2]. According to the study, Egypt ranks 7^th^ in the World for obesity.

Obesity is associated with a greater risk of disability or premature death due to type 2 diabetes mellitus, cardiovascular diseases (such as stroke and coronary heart disease), gall bladder disease, certain cancers (endometrial, breast, prostate, colon) and non-fatal diseases including gout, respiratory conditions, gastro-esophageal reflux disease, osteoarthritis and infertility [3]. Obesity also carries serious implications for psychosocial health, mainly due to an evolving societal prejudice against fatness.

It has been reported that polymorphisms in genes controlling appetite and metabolism are associated with susceptibility to obesity (when sufficient food is available). More than 41 such genes and many variations in theses genes have been shown to be linked to the development of obesity when the environment is permissive [4]. However, genetics is but part of a complex, involving societal, environmental and individual psychological factors.

In Man insoluble lipids are transported in blood as lipoprotein complexes with one or more apolipoproteins. Low-density lipoprotein (LDL) is 75% lipid (cholesterol and cholesteryl esters) and 25% protein. A high level of LDL is a risk factor for cardiovascular disease [5]. ApolipoproteinB (ApoB) plays a central role in lipid metabolism as the main protein component of VLDL and LDL. It also serves as the ligand for removal of LDL from the circulation by LDL-receptor-mediated endocytosis [6]. ApoB is the protein primarily responsible for transporting cholesterol in LDL to tissues. Although it is uncertain what functional role ApoB plays in LDL, it is absolutely required for its formation. ApoB on the LDL particle acts as a ligand for LDL receptors in various cells throughout the body. High plasma ApoB levels are a factor contributing to plaques that cause atherosclerosis [7]. Variants of the *apoB* gene may therefore be involved in the pathogenesis of obesity.

The gene coding for human ApoB [ID: 338] is 43 kb in length, located on the short arm of Chromosome 2 (2p23-p24) [8]. Several single nucleotide polymorphisms in the *apoB* gene have been associated with variation in lipid levels and obesity. These include XbaI (X; 9), EcoRI (R; 10) and MspI (M; 11). A single nucleotide transition (C#T) at position 7673 creates a restriction site (ACC → ACT) for XbaI enzyme characterizing the ‘X+’ allele, whereas no change determines the ‘X-‘ allele. The changed amino acid at codon 2488 of exon 26 is silent, Thr2488Thr (T2488T)’ [7]. This polymorphism has been found to be associated with inter-individual variability of lipid levels, but the results are inconsistent [9]. Epidemiological studies have shown an association between *apoB* gene polymorphisms and generalized and regional obesity and an increase in various lipoprotein subfractions (total cholesterol [TC], low density lipoprotein cholesterol [LDL-C], triglyceride [TG]), and atherosclerosis [10-12]. Most of these studies have been carried out in Caucasian populations. It has been reported that the X+ allele of the Apo B gene is a risk factor for the development of cholelithiasis (gallstones) in Chinese patients [13]. However, in a Mexican population study no association was shown to exist between the *apoB* XbaI polymorphism and cholelithiasis [14]. A significant association of *apoB* XbaI gene polymorphism with obesity and serum lipid levels [12] has been documented, but contradictory results are also available. Misra *et al.* [15] reported that *apoB* XbaI gene polymorphism did not associate with obesity.

In this study we have attempted to correlate *apoB* XbaI gene polymorphisms with obesity and lipid profile in obese and non-obese Egyptian subjects.

## Subjects and methods

### Subjects

178 obese individuals aged 19 to 60 years were recruited in Egypt. 178 normal Egyptian individuals with no diagnosis of obesity, and no family history of obesity were also recruited. The enrolled subjects were referred to the National Research Center clinic during the period 2012 – 2013. Their ages and sex were matched with the obese group. All subjects gave their informed consent and the protocol was approved by the National Research Center of Egypt.

All subjects gave detailed clinical histories, and measurements included: height, weight, BMI, and waist-to-hip ratio (WHR). BMI was calculated as weight in kilograms divided by the square of height in metres and obesity was defined as BMI ≥ 25 kg/m^2^. Only non-smoking, normotensive (systolic < 130 mmHg; diastolic < 85 mmHg) and non-diabetic (fasting blood sugar < 110 mg/dl) subjects were included. Subjects were considered as normal if they fall in normal ranges for various parameters. For example, leptin = 2–11 mg/ml, serum insulin = 0–30 μU/ml. A history of coronary artery disease, neoplasia, congenital and mental disorders, and endocrine disorders such as myxoedema and Cushing syndrome were defined as additional criteria for exclusion from this study.

### Anthropometry measurements

Body weight was measured to the nearest 0.1 kg, and height was measured to the nearest 0.01 m [16]. The waist circumference was measured half way between the lower rib and iliac crest, the hip circumference was measured over the widest part in the gluteal region, and the WHR was calculated [17].

### Sample collection

Blood (10 ml) was collected without anticoagulant after a 12-h fast and the serum was used for the determination of lipids from all subjects. The collected blood for serum preparation was kept in an upright position at room temperature for 30 minutes to allow collecting. Centrifugation for 15 min 2000 RCF with no braking followed. The supernatant (serum) was carefully aspirated at room temperature and transferred into tubes. Unturbid samples were aliquoted into cryovials and stored at – 80°C for subsequent analysis. Blood was collected in vacutainer tubes with EDTA for analysis of DNA. Genomic DNA was extracted from peripheral blood leukocytes [18].

### Biochemical parameters investigations

Five lipid parameters (Total Cholesterol, Triglycerides, High Density Lipoprotein, Low Density Lipoprotein, and Very Low Density Lipoprotein) were measured in obese and non-obese subjects. TC and TG were measured by enzymatic colorimetric tests [19,20]. HDL-cholesterol concentrations were determined by enzymatic assay after phosphotungstic acid and magnesium precipitation [21]. LDL-cholesterol was calculated according to Friedewald’s formula and VLDL-cholesterol was calculated using the formula TG/5 [22]. Fasting blood sugar was assayed by glucose oxidase-peroxidase) method [23]. All values were measured in mg/dl. Serum leptin and insulin was assayed to exclude monogenic obesity. In addition, estimation of FT3, FT4, TSH, growth hormone, FSH and LH was done to exclude endocrine causes.

### DNA isolation and genotyping

Genomic DNA extraction was done using salting out methodology [18]. The isolated DNA was of good quality (absorbance 260 nm/280 nm, ratio > 1.75). The desired segment was amplified by PCR using the *apoB* XbaI protocol [23] with the respective primers (Fermantas Inc., USA): 5′- GGAGAC TATTCAGAAGCTAA-3′ as forward primer and 5′-GAAGAGCC TGAAGACTGACT-3′ as reverse primer. The amplified products were restriction digested using XbaI and digested products were electrophorized on 1.5% agarose gel containing ethidium bromide (1%). After the run was completed, the gel was visualized under UV light. The genotypes for all samples were reassessed twice to confirm the results and ensure reproducibility. Some uncertain genotypes were validated by purifying the PCR products using GeneJET gel extraction and DNA cleanup kit (Thermo Fisher Scientific Inc. USA) and were directly sequenced on ABI Prism 310 Automated Sequencer, using the ABI Prism Big Dye Terminator Cycle Sequencing Ready Reaction Kit (PE Applied Biosystems).

### *apoB* gene XbaI polymorphism status

The following nomenclature has been used to specify the genotypes at *apoB* XbaI: **X-/X-** (wild-type (CC) or homozygous absence of restriction site), **X-/X+** ((CT) heterozygous absence of restriction site deletion); and **X+/X+**: (rare-type (TT) or homozygous presence of restriction site).

### Statistical analysis

All the statistical calculations for biochemical factors were performed using IBM SPSS Statistics v. 11.5 for Windows. For each variable, the values were expressed as mean ± SD. Data was evaluated by t test and one-way analysis of variance. Allele and genotypic frequencies for *apoB* XbaI was calculated with the gene counting method. Hardy-Weinberg equilibrium, as well as comparison of the categorical data i.e., different *apoB* genotypes among non-obese controls and obese subjects were made by the χ^2^ test. Odd’s ratios were calculated with a 95% confidence interval limit using 2 × 2 contingency table and p-value < 0.05 was used as the level of significance.

## Result

356 subjects comprising obese (84 male and 94 female) and non-obese (91 male and 87 female) adults were evaluated. The mean BMI ( kg/m^2^) of the obese and non-obese subjects was 29.91 ± 3.56 and 20.37 ± 2.54, respectively. The WHR (p-value < 0.001), Systolic (*p-value* < 0.001) and diastolic (*p-value* < 0.002) blood pressures, though in normal range, were significantly higher in obese subjects as compared to non-obese subjects. Fasting blood glucose (mg/dl) was significantly higher in obese subjects as compared to non-obese subjects. TC, TG, LDL, and VLDL were also higher (*p-value* ≤ 0.001) in obese subjects as compared to non-obese subjects. On the contrary, HDL was significantly higher (p-value < 0.001) in non-obese as compared to obese subjects Table 1.

**Table 1:** **Obesity and lipid parameters in the Egyptian cohort**

**Parameter**	**Obese (n = 178)**	**Non-obese (control; n = 178)**	**p-value**
WHR	0.95 ± 0.03	0.82 ± 0.04	<0.001
SBP (mmHg)	123.89 ± 11.91	116.18 ± 9.25	0.001
DBP (mmHg)	81.82 ± 8.85	78.27 ± 7.84	0.002
TC (mg/dl)	264.52 ± 29.78	169.84 ± 31.23	<0.001
HDL-C (mg/dl)	31.48 ± 1.56	41.68 ± 4.78	<0.001
VLDL-C (mg/dl)	30.86 ± 10.43	20.86 ± 4.13	<0.001
TG (mg/dl)	197.35 ± 21.56	103.76 ± 14.87	<0.001
LDL-C (mg/dl)	175.12 ± 36.75	106.64 ± 34.96	<0.001

WHR: Waist-to-hip ratio; SBP: Systolic blood pressure; DBP: Diastolic blood pressure; TC: Total cholesterol; HDL-C: High density lipoprotein cholesterol; LDL-C: Low density lipoprotein cholesterol; VLDL-C: Very low density lipoprotein cholesterol; TG: Triglyceride.

### Association of *apoB* XbaI gene polymorphism (X) with obesity

Initial genotype analysis indicated the presence of the XbaI polymorphism (X) in the Egyptian population and this was confirmed by direct sequencing. The genotype frequencies did not deviate significantly from the Hardy-Weinberg expectations in the obese (χ^2^ = 0.09, p = 0.77) and non-obese control subjects (χ^2^ = 0.025, p = 0.47). Analysis did not reveal a positive association between the *apoB* XbaI gene polymorphism (X) and obesity-related phenotypes. The observed allele frequencies were X− (C), 70.2% versus 67.1%; and X+ (T), 32.3% versus 32.9% in obese and non-obese subjects. The frequencies of X + X+ (TT) genotype did not differ significantly in obese and non-obese subjects. Similarly, frequency of X+ (T) allele was not significantly different in the two groups Table 2. In obese and in non-obese subjects, none of the obesity related parameters (especially TC, TG, LDL, HDL, and VLDL) were associated with genotypes of *apoB* Table 3. The *apoB* X+ allele did not show any statistically significant difference with clinically profiled obese or non-obese subjects Table 3. Lipid levels (TC, TG, LDL, HDL, and VLDL) were significantly different in obese and non-obese subjects. No significant association was demonstrated between genotypes of *apoB* XbaI gene polymorphism (X+ carrier and X+ non-carrier) and lipid profile in obese and non-obese subjects Table 3.

**Table 2:** ***apoB*** **XbaI Polymorphism analysis (X-/X- (CC), X-/X+ (CT), X+/X+ (TT) and X- (C) and X+ (T) alleles) in obese subjects (n = 178) and non-obese controls (n = 178)**

	***apoB*** **genotype**			***apoB*** **allele**	
	**CC**	**CT**	**TT**	**C**	**T**
Non-obese (%)	88	63	27	151	117
	(49.4)	(35.4)	(15.2)	(67.1)	(32.9)
Obese (%)	97	56	25	250	115
	(54.5)	(31.5)	(14)	(70.2)	(32.3)
p-value	-	0.200	0.427	-	0.250
OR	1.0	0.768	0.746	1.0	0.727
(95% CL)	(reference)	(0.456 – 1.23)	(0.374 – 1.64)	(reference)	(0.538 – 1.276)

OR = Odd ratio; 95% CI = confidence interval at 95%.

**Table 3:** **Association of** ***apoB*** **XbaI gene polymorphism (X) with obesity related parameters**

**Parameter**	**TC (mg/dl)**	**HDL-C (mg/dl)**	**HDL-C (mg/dl)**	**TG (mg/dl)**	**TG (mg/dl)**
**Obese subjects**					
**X-/X- (CC)**	253.50 ± 5.67	32.12 ± 3.54	27.74 ± 5.34	179.75 ± 8.34	178.13 ± 6.75
**X-/X+ (CT)**	245.38 ± 8.91	31.48 ± 2.58	29.74 ± 2.41	178.51 ± 3.42	175.24 ± 5.67
**X+/X+ (TT)**	253.3 ± 4.15	31.38 ± 2.11	32.53 ± 1.31	181.32 ± 3.58	169.36 ± .32
**P value**	0.973	0.412	0.736	0.231	0.872
**X+ carrier**	273.86 ± 5.85	32.51 ± 4.26	28.98 ± 3.57	147.64 ± 3.82	212.45 ± 6.67
**X+ non-carrier**	273.54 ± 4.82	31.83 ± 3.68	28.57 ± 1.94	146.56 ± 4.85	211.95 ± 4.74
**P value**	0.428	0.841	0.263	0.471	0.581
**Non-obese subjects**					
**X-/X- (CC)**	172.78 ± 9.73	43.24 ± 5.93	21.00 ± 3.35	104.6 ± 6.59	104.35 ± 6.69
**X-/X+ (CT)**	176.75 ± 6.15	42.83 ± 5.47	19.63 ± 3.14	98.14 ± 5.67	105.94 ± 6.51
**X+/X+ (TT)**	171.53 ± 4.38	42.03 ± 5.12	21.25 ± 2.89	106.56 ± 4.38	107.13 ± 4.87
**P value**	0.872	0.752	0.234	0.213	0.842
**X+ carrier**	219.89 ± 4.69	38.94 ± 3.59	25.57 ± 2.73	123.54 ± 3.35	155.51 ± 4.42
**X+ non-carrier**	206.18 ± 6.72	41.30 ± 2.31	23.93 ± 3.74	115.85 ± 6.78	146.16 ± 9.79
**P value**	0.062	0.074	0.323	0.32	0.061

TC: Total cholesterol; HDL-C: High density lipoprotein cholesterol; LDL-C: Low density lipoprotein cholesterol; VLDL-C: Very low density lipoprotein cholesterol; TG: Triglyceride.

## Discussion

We have examined the genotypic and allelic frequencies and the effect of *apoB* XbaI polymorphism (X) on obesity and lipid profile in well-defined Egyptian obese and non-obese populations. The *apoB* XbaI polymorphism (X) was selected for the present study by virtue of its documented association with obesity and dyslipidemia elsewhere [12]. Various studies have shown an association between the Apo B gene polymorphisms with lipoprotein subfractions (TC, LDL-C, and TG) [21-23]. However, here we showed that there was no significant difference in the genotype and allele frequencies of *apoB* XbaI polymorphism between obese and non-obese subjects. Saha *et al*. [14] also reported no significant difference in allelic frequencies of XbaI gene polymorphism of the *apoB* gene. In addition, another study did not show considerable association of APOB XbaI gene polymorphism with obesity and lipid profile in north Indians [24].

In the present study, a comparison of clinical variables was also done in relation to the genotypes of the *apoB* XbaI gene in obese and non-obese subjects but no association was found with serum lipid levels. Our results were in agreement with a previous study, [25] while Saha et al. [19] reported no association between the apoB XbaI gene and serum lipids levels.

It may be that apoB XbaI polymorphism exhibits population specific variation, which may be due to gene and environment interactions. apoB XbaI polymorphism does not lead to changes in the amino acid sequence and cannot be implicated at structure level. It is possible that some other polymorphism in its vicinity might be present, which is in linkage disequilibrium with apoB XbaI polymorphism and accountable for the observed association with obesity and lipid levels in other studies. The effect of apoB XbaI polymorphism on the lipid levels is due to linkage disequilibrium with ins/del polymorphism, which causes an amino acid change in the signal peptide of the apoB gene. Saha et al. [11] showed a strong disequilibrium between ins/del polymorphism and the XbaI polymorphism of apoB gene. Among Tunisian population the XbaI polymorphism effect was only observed for triglyceride in men. This rare study indicated an influence of XbaI polymorphism of Apo B gene on serum total-cholesterol, triglycerides and apolipoprotein A1 concentrations [26]. Moreover, gene–environment interaction may also responsible for the inconsistency of data due to differences in the diet and lifestyle of populations of various part of the world. It has been reported that overweight subjects from the Bichat Hospital in Paris with the Del allele of the apo B signal peptide polymorphism were more susceptible to high LDL cholesterol levels but their LDL cholesterol responds well to diet. This study shed light about the importance of the interaction between genes and nutritional environment in the determination of the lipid levels [27].

## Conclusion

In summary, there was no evidence of association of apoB polymorphisms (XbaI) with obesity and serum lipid levels in the studied Egytian cohorts. There may be several reasons for the differences observed in the various studies. The populations studied in different studies may have been genetically different. In view of the strong associations reported in some studies, we suggest larger sample sizes involving differing ethnicities may be needed to study the association of XbaI polymorphism with obesity [9,10,18].
